# Recurrent membranous nephropathy with a possible alteration in the etiology: a case report

**DOI:** 10.1186/s12882-021-02457-0

**Published:** 2021-07-06

**Authors:** Ayumi Matsumoto, Isao Matsui, Keiji Mano, Hitoshi Mizuno, Yusuke Katsuma, Seiichi Yasuda, Karin Shimada, Kazunori Inoue, Takashi Oki, Tadashi Hanai, Keiko Kojima, Tetsuya Kaneko, Yoshitaka Isaka

**Affiliations:** 1 Department of Nephrology, Osaka University Graduate School of Medicine, 2-2 Yamada-oka, Suita, Osaka, 565-0871 Japan; 2 Department of Nephrology, Daini Osaka Police Hospital, 2-6-40, Karasuga-tsuji, Tennoji, Osaka, 543-8922 Japan; 3 Department of Urology, Mimihara General Hospital, 4-465, kyowacho, Sakai-ku, Sakai, Osaka, 590-8505 Japan; 4 Department of Urology, Daini Osaka Police Hospital, 2-6-40, Karasuga-tsuji, Tennoji, Osaka, 543-8922 Japan; 5 Department of Pathology, Daini Osaka Police Hospital, 2-6-40, Karasuga-tsuji, Tennoji, Osaka, 543-8922 Japan

**Keywords:** Membranous nephropathy, Cancer, Thrombospondin type-1 domain-containing 7A

## Abstract

**Background:**

Phospholipase A2 receptor 1 (PLA2R1) and thrombospondin type-1 domain-containing 7A (THSD7A) are the two major pathogenic antigens for membranous nephropathy (MN). It has been reported that THSD7A-associated MN has a higher prevalence of comorbid malignancy than PLA2R1-associated MN. Here we present a case of MN whose etiology might change from idiopathic to malignancy-associated MN during the patient’s clinical course.

**Case presentation:**

A 68-year-old man with nephrotic syndrome was diagnosed with MN by renal biopsy. Immunohistochemistry showed that the kidney specimen was negative for THSD7A. The first course of corticosteroid therapy achieved partial remission; however, nephrotic syndrome recurred 1 year later. Two years later, his abdominal echography revealed a urinary bladder tumor, but he did not wish to undergo additional diagnostic examinations. Because his proteinuria increased consecutively, corticosteroid therapy was resumed, but it failed to achieve remission. Another kidney biopsy was performed and revealed MN with positive staining for THSD7A. PLA2R1 staining levels were negative for both first and second biopsies. Because his bladder tumor had gradually enlarged, he agreed to undergo bladder tumor resection. Pathological examination indicated that the tumor was THDS7A-positive bladder cancer. Subsequently, his proteinuria decreased and remained in remission.

**Conclusions:**

This case suggests that the etiology of MN might be altered during the therapeutic course. Intensive screening for malignancy may be preferable in patients with unexpected recurrence of proteinuria and/or change in therapy response.

**Supplementary Information:**

The online version contains supplementary material available at 10.1186/s12882-021-02457-0.

## Background

Idiopathic membranous nephropathy (MN) is an immune disease associated with the immune complex. Phospholipase A2 receptor 1 (PLA2R1) and thrombospondin type-1 domain-containing 7A (THSD7A) are recognized as target antigens of MN [1, 2]. Moreover, it is reported that the prevalence of malignancy is higher in patients with THSD7A-associated MN than PLA2R1-associated MN [3]. However, the pathophysiological relationship between the generation of anti-THSD7A antibody and malignancy is unclear. We present a case of MN, in which the etiology might be changed during the clinical and therapeutic course.

## Case presentation

**Fig. 1:**
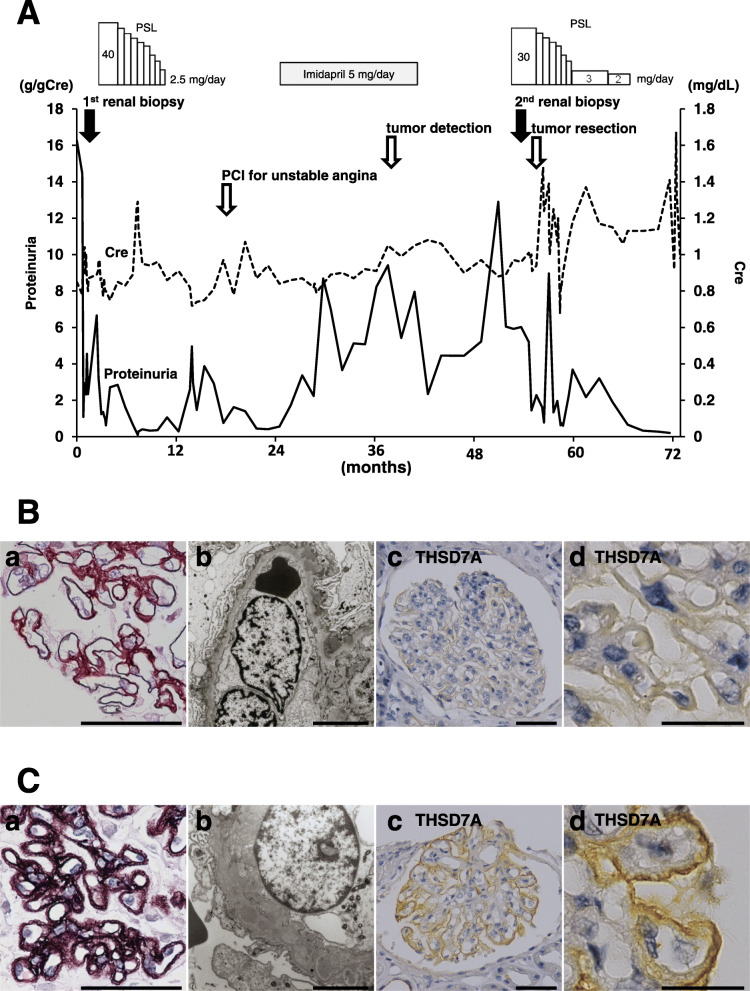
Clinical findings of this case are shown. **A** Proteinuria and serum creatinine are shown. **B** (a) Periodic acid methenamine silver (PAM)-stained kidney sections observe by light microscopy and (b) ultrathin sections observe by electron microscopy reveal that the patient at age 68 has membranous nephropathy stage II. (c and d) Immunohistochemical analysis shows that the specimen of the first renal biopsy is negative for THSD7A. **C** (a) PAM-stained kidney sections and (b) ultrathin sections reveal that the patient at 72 years has membranous nephropathy stage III. (c and d) Immunohistochemistry shows that the specimen of the second renal biopsy is positive for THSD7A. (scale bars: (a) 50 μm, (b) 5 μm, (c) 50 μm, and (d) 20 μm) Abbreviations: PCI, percutaneous coronary intervention; PSL, prednisolone

**Fig. 2:**
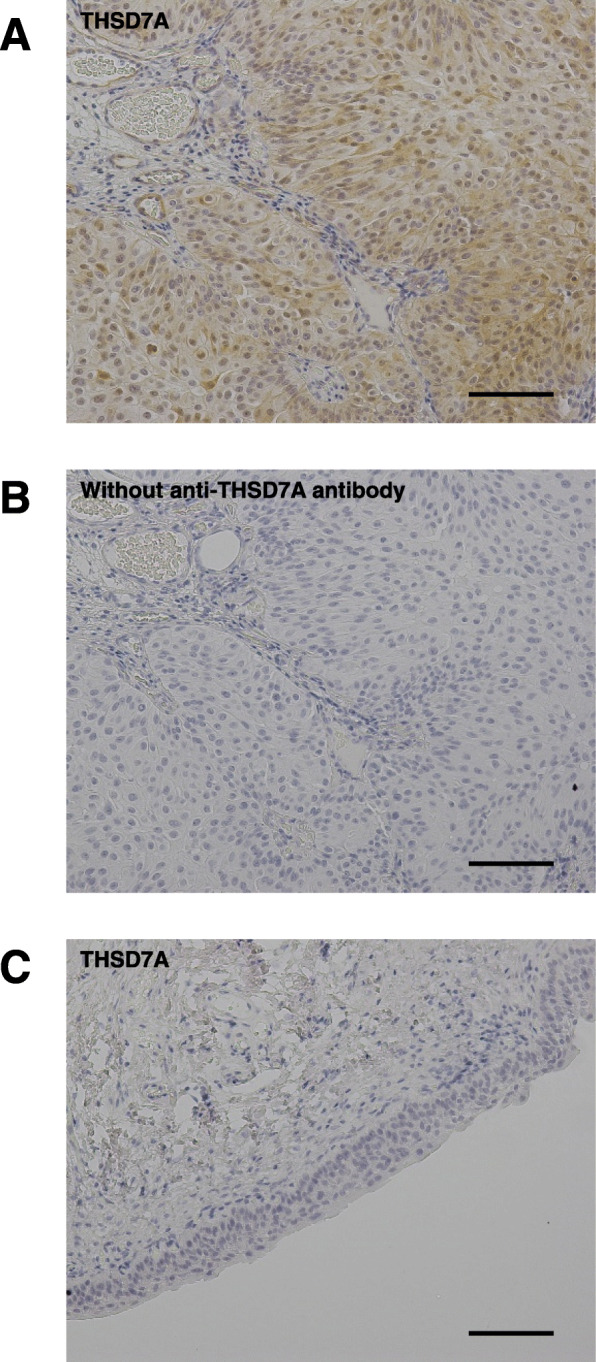
**A** Resected bladder cancer is positive for THSD7A. **B** Immunohistochemistry without anti-THSD7A antibody yields no positive signal. **C** Normal portion of the transitional epithelia is negative for THSD7A (scale bars: 100 μm)

A 68-year-old man who had hypertension for 14 years was transferred to our facility for the management of nephrotic syndrome with proteinuria at 16.3 g/gCre (Fig. 1A). On admission, he was conscious, and his lower extremities were edematous. His serum creatinine, urea nitrogen and albumin were 0.85 mg/dL, 8 mg/dL and 2.8 g/dL, respectively. To investigate his pathophysiology, a renal biopsy was performed. It revealed that his nephrotic syndrome was caused by stage II MN (Fig. 1B-a,b). Immunohistochemistry showed that the kidney specimen was negative for THSD7A (Fig. 1B-c, d). We shared the diagnosis and therapeutic strategy with him. Based on his agreement, subsequent corticosteroid therapy successfully suppressed his proteinuria to 0.6 g/gCre within 6 months. Every outpatient care visit, he had good adherence to the medication. During the course of corticosteroid therapy, he did not experience any unanticipated event, including abnormal glucose tolerance and exacerbation of hypertension. The remission of proteinuria led to the termination of corticosteroid therapy. At age 69, recurrence of proteinuria (up to 4.9 g/gCre) was observed; however, we hesitated to administer corticosteroid because of unstable angina. At 71 years of age, abdominal ultrasonography revealed a urinary bladder tumor 5 mm in diameter. However, he did not want to undergo further examinations at that time. At 72 years of age, his proteinuria increased up to 12.9 g/gCre. Therefore, we resumed corticosteroid therapy, but remission was not achieved. We performed another renal biopsy, which showed that the patient had MN whose stage has progressed from II to III (Fig. 1B and C). Immunohistochemical staining revealed that the kidney sections were positive for THSD7A in the second biopsy, but not in the first biopsy (Fig. 1B-c, d and C-c, d), while PLA2R1 staining levels were negative for both the first and second biopsies. (Supplemental Figure S1). Because the size of the bladder tumor had increased to 10 mm in diameter, we resected the tumor. Pathological findings indicated that the tumor diagnosis was THSD7A -positive bladder cancer (Fig. 2). Serum levels of anti-PLA2R1 and anti-THSD7A antibodies were not measured in this case due to lack of stored serum samples. Although proteinuria temporally increased after the surgery, the proteinuria level decreased to 0.3 g/gCre within 12 months following the tumor resection.

## Discussion and conclusion

Herein we present a case of THSD7A-associated MN. PLA2R1 and THSD7A are now recognized as target antigens of MN [1, 2]. The prevalence of serum anti-PLA2R1 and anti-THSD7A antigens are reported to be 52–98% and 2.5–9.1%, respectively [4]. The etiology of THSD7A-associated MN is reportedly different from that of PLA2R1-associated MN in terms of malignant tumor involvement. The prevalence of malignant tumors was 5.4% in PLA2R1-positive MN and 20–33% in THSD7A-associated MN [3].

In this case, though the first kidney biopsy showed normal immunohistochemical staining for THSD7A, the second biopsy showed enhanced staining. Furthermore, the comorbid bladder tumor tissue was positive for THSD7A, while surrounding normal bladder tissue, obtained at the time of cancer resection surgery, showed a negative stain for THSD7A. Although we could not determine serum levels of anti-PLA2R1 and anti-THSD7A antibodies due to lack of stored serum samples, various studies have revealed that renal immunostaining patterns of PLA2R1 and THSD7A in the presence of circulating anti-PLA2R and anti-THSD7A antibodies are different from those in the absence of these antibodies [2, 5–7] The findings of the current case suggest that the incipient nephrotic syndrome was led by idiopathic MN, and the recurrent nephrotic syndrome was led by malignancy-associated MN. The different responses to corticosteroid therapies in the incipient and the recurrent nephrotic syndrome also suggest that the etiology of MN in this case might be altered during the therapeutic course. However, there remains a possibility that both the incipient and the recurrent MN were caused by bladder tumor. The production of more THSD7A antigens in the gradually enlarged bladder tumor might cause the enhanced staining of THSD7A in the second kidney biopsy. The analysis of IgG-subclasses would help to distinguish primary MN from secondary MN. However, we could not determine IgG subclasses because fresh-frozen kidney tissues are not stored. It has been reported that the dominant IgG subclasses did not change over time in recurrent MN in transplanted kidney, suggesting that the MN etiology under transplantation setting might not change [8]. Further studies are required to clarify whether cancer can alter the etiology of recurrent MN.

Based on similar clinical findings in previously published reports, the causal relationship between malignant tumor and MN has been discussed [3, 9]. We have reported that vascular endothelial growth factor-A (VEGF-A) induces THSD7A expression in human umbilical vein endothelial cells [10]. Malignant tumors are known as neovascularization-rich tissue, where VEGF-A plays an essential role in its pathogenesis [11]. This fact may explain the positive stain for THSD7A only in the tumor tissue but not in the normal tissue in this case. Although the precise mechanisms of THSD7A-associated MN remain unclear, there might be a hypothesis that VEGF-A induces THSD7A expression as an immunogenic protein at the tumor loci. Further investigation is required to warrant this hypothesis.

To the best of our knowledge, this is the first report showing that a malignant tumor might alter the etiology of recurrent MN. The therapeutic course supports the hypothesis that THSD7A-positive bladder cancer was responsible for developing THSD7A-positive MN. Intensive cancer screening may have to be performed upon a finding of THSD7A-positive MN. Furthermore, it might be advisable to perform repeat renal biopsy when unexpected recurrence of proteinuria occurred or a change in therapeutic response was observed.

## Methods

Biopsy samples were fixed in a 10% neutral buffered formalin solution, dehydrated in an increasing ethanol concentration solution series, and embedded in paraffin. Paraffin-embedded sections were stained with periodic acid methenamine silver using standard procedures. Antibodies against specific molecules were obtained as PLA2R1 (HPA012657; Sigma-Aldrich, St. Louis, MO, USA) and THSD7A (HPA000923; Sigma-Aldrich, St. Louis, MO, USA). Immunohistological staining was performed on freshly-cut specimens using standard methods [12–19].

## Supplementary Information

**Additional file 1: Supplemental Figure S1.** Immunohistochemical analysis for PLA2R1 on (A) a specimen from the first biopsy and (B) a specimen from the second biopsy. (scale bars: (a) 50 μm and (b) 20 μm).

## Data Availability

All data analyzed during this study are included in this manuscript.

## Abbreviations

MN: Membranous nephropathy
PLA2R1: Phospholipase A2 receptor 1
THSD7A: Thrombospondin type-1 domain-containing 7A
VEGF-A: Vascular endothelial growth factor-A
