# Supplementary figures and images for: Recurrent membranous nephropathy with a possible alteration in the etiology: a case report
Source: BMC Nephrol. 2021 Jul 6;22:253. doi: 10.1186/s12882-021-02457-0 (PMC8258946; doi:10.1186/s12882-021-02457-0)

Supplemental Figure S1

**A**

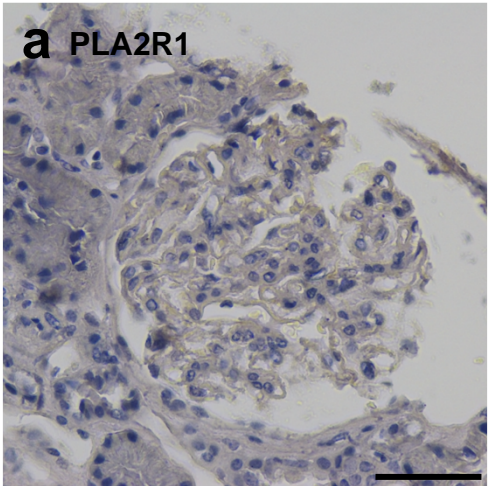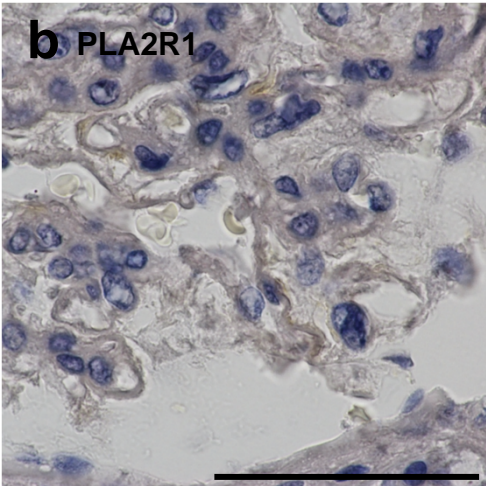

**B**

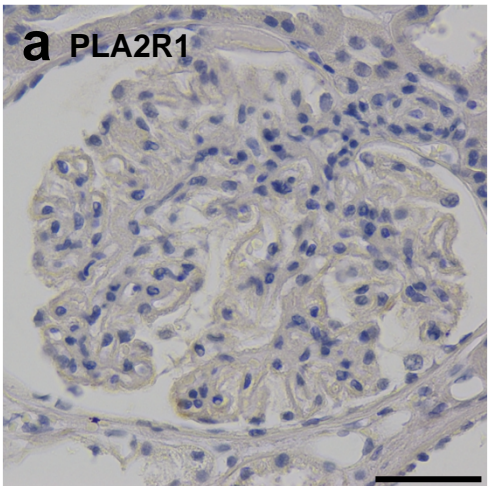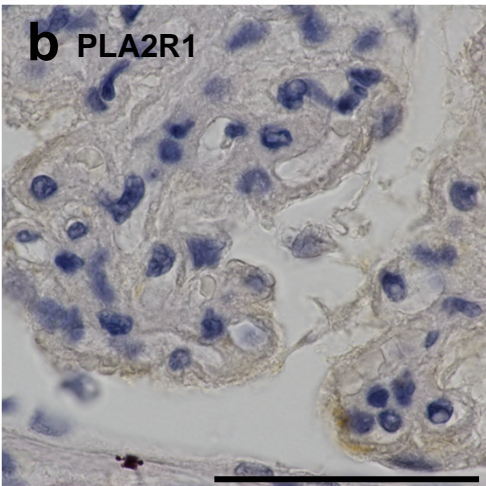

Supplement: Supplementary file 1 — Additional file 1: Supplemental Figure S1. Immunohistochemical analysis for PLA2R1 on (A) a specimen from the first biopsy and (B) a specimen from the second biopsy. (scale bars: (a) 50 μm and (b) 20 μm). [file 12882_2021_2457_MOESM1_ESM.pdf]
